# Dogs’ Gazing Behavior to Humans Is Related to Their Liveliness, Aggressiveness, and the Emotional Comfort They Provide

**DOI:** 10.3390/ani15040483

**Published:** 2025-02-08

**Authors:** Eniko Kubinyi, Andrea Sommese, Márta Gácsi, Ádám Miklósi

**Affiliations:** 1 Department of Ethology, Eötvös Loránd University, 1117 Budapest, Hungary; andrea.sommese@vetmeduni.ac.at (A.S.); marta.gacsi@ttk.elte.hu (M.G.); adam.miklosi@ttk.elte.hu (Á.M.); 2 MTA-ELTE “Momentum” Companion Animals Research Group, 1117 Budapest, Hungary; 3 HUN-REN-ELTE Comparative Ethology Research Group, 1117 Budapest, Hungary

**Keywords:** dog personality, interspecific communication, gazing behavior, human–animal relationship, ambiguous situation

## Abstract

Gazing toward humans is fundamental in dog–human communication. This study aimed to characterize companion dogs based on their gazing behavior and to explore the potential links between gazing, personality (liveliness, confidence, aggressiveness, and attachment), emotional comfort provided to the owners, and breed. We observed 171 dogs in an ambiguous situation involving a moving stimulus, a remote-controlled toy car and gathered additional data with questionnaires. Our behavior analysis, based on gaze frequency directed at the owner and experimenter, identified four distinct clusters of dogs: low gazers, experimenter-focused gazers, owner-focused gazers, and frequent gazers. We found that experimenter-focused gazers exhibited lower levels of liveliness than frequent gazers and were reported to be less aggressive than low gazers and frequent gazers. Owner-focused gazers provided more emotional comfort to their owners than low gazers. Regarding breeds, 56.5% of German shepherd dogs were low gazers, compared to only 5.3% of golden retrievers. Age, sex, neutered status, and approaching the stimulus were not associated with cluster membership. These results highlight the complexity of dog–human communication, suggesting that personality, emotional factors, and breed shape how dogs seek guidance and support from humans or express anxiety.

## Simple Summary

Dogs use gazing behavior to communicate with humans. This study explored how family dogs differ in their gazing behavior in an uncertain situation and how these differences relate to personality, emotional comfort, and breed. We observed dogs responding to a novel stimulus—a remote-controlled toy car—and analyzed how often they looked at their owner or the experimenter in one minute. Based on this behavior, dogs were grouped into four clusters. Dogs that gazed 6–7 times at each human partner were rated as less aggressive than those that gazed only 2–3 times. They were also perceived as less lively than dogs that gazed 10–12 times at each partner. Additionally, dogs gazing more frequently at the owner than at the experimenter were reported to provide more emotional comfort to their owners, such as making them feel loved. Breed differences also emerged, with German shepherd dogs more likely to be low gazers compared to golden retrievers. Interestingly, a dog’s age, sex, or willingness to approach the toy car did not affect their gazing behavior. These findings deepen our understanding of how dogs communicate with humans and the factors influencing this behavior, which can improve training, strengthen bonds, and enhance canine welfare.

## 1. Introduction

Social referencing allows individuals to rely on the emotional displays of others to evaluate situations, enabling them to avoid potential mistakes. This ability is particularly advantageous for young or inexperienced individuals (e.g., [1,2]). In humans, social referencing has been extensively studied and is evident even at a very young age. For instance, toddlers and infants look at caregivers or other informants and adjust their behavior based on the emotional signals they receive [3,4,5].

This behavior has also been observed in primates. Itakura [1] demonstrated that captive chimpanzee infants (*Pan troglodytes*) engaged in social referencing by frequently gazing at and returning to their mothers in the presence of a novel object. Similar findings were reported in nursery-reared chimpanzees, who adjusted their behavior based on emotional cues from familiar caregivers [6]. In Barbary macaques (*Macaca sylvanus*), Roberts et al. [2] noted that older infants engaged in social referencing more frequently than younger ones, albeit with less reliance on the mother when she could not see the object.

Dogs, as a domesticated species, also exhibit sensitivity to human cues, including gaze direction and body orientation (e.g., [7,8,9,10]). Research shows that dogs frequently gaze at humans in various contexts [11,12] and sometimes do so more than other species [13,14,15]. In problem-solving scenarios, this behavior is often interpreted as an attempt to initiate communicative interactions [16,17].

There is substantial variability in dogs’ gazing behavior, influenced by multiple factors. Artificial selection for specific roles (e.g., companionship or working purposes) has shaped their ability to communicate with humans [18,19,20,21], though some breeds (e.g., Czechoslovakian wolfdogs) remain less human-oriented [22,23]. Age is potentially another factor, as it has been reported to influence gazing behavior in unsolvable tasks, with adult dogs gazing sooner and longer than puppies [24] and older dogs sooner, longer, and more frequently than younger ones [20]. However, the effect of age is not clear because in a test where dogs were rewarded for establishing eye contact with an experimenter, older dogs gazed with longer latency at humans than younger ones [19], and in a gaze-following test, age had no effect except that the youngest and oldest age groups were more distractible, looking more frequently at humans [25]. Training might also play a role; for example, in problem-solving tests, trained dogs are better problem-solvers, while untrained dogs look more at the human partners [26], but agility-trained dogs gaze at humans more frequently than search-and-rescue or untrained dogs [12]. In an attention test, assistance dogs show longer and less frequent gazes towards the owner than untrained dogs, with intermediate values for agility dogs [27]. Sex differences have also been observed, with female dogs of unspecified breed function spending more time gazing at images of human and dog heads or objects that “magically” changed size compared to males [28,29]. Personality traits further modulate gazing behavior. Sociable dogs exhibit more persistent human-directed gazing [30], while anxious dogs tend to look at humans longer during unsolvable tasks [31]. Notably, most studies focus on gazing behaviors in problem-solving tasks, leaving gaps in understanding dogs’ gazing patterns in other contexts, such as ambiguous or stressful situations. Finally, studies on ambiguous contexts have shown that dogs’ relationships with their owners might also influence gazing behavior. For example, in the presence of a novel object, dogs frequently gaze at their owners and are more likely to approach the object when the owner provides positive emotional cues [32,33]. Similar patterns have been observed in situations involving social threats [34].

Despite these findings, the relationship between dogs’ personality traits, the emotional comfort they provide to owners, and their gazing behavior in ambiguous contexts remains underexplored. This study aims to fill this gap by examining the link between dogs’ gazing behavior towards social partners (the owner, the person living in the same household primarily responsible for the privately owned cat or dog, and a stranger, an unfamiliar female experimenter), their personality traits, and the emotional comfort they provide. Specifically, we used a remote-controlled toy car as an ambiguous stimulus to induce moderate stress [35] and observed dogs’ gazing patterns and approach behavior. Additionally, we investigated associations between dogs’ approach behavior, personality traits (measured using the Budapest Canine Personality Survey [36]), and the emotional comfort they provide to their owners (assessed via a questionnaire). We hypothesized that (1) dogs can be grouped by their gaze frequency patterns toward the partners; (2) untrained dogs would gaze less at both partners than trained dogs; (3) older dogs would gaze more at both the owner and the stranger compared to younger dogs; (4) female dogs would gaze more frequently at the partners than males; (5) purebred dogs look at humans more than mixed-breed dogs; (6) dogs with different gazing frequency differ in their personality traits; (7) dogs looking more often at the owner than the experimenter provide more emotional comfort to their owners; and (8) dogs approaching the ambiguous stimulus (remote-controlled toy car) gaze less at the partners.

## 2. Materials and Methods

### 2.1. Subjects

A sample of 171 dogs (mean age = 4.36 ± 2.94, min = 0.6, max = 14 years; 57% males, of which 33% were neutered; 43% females of which 49% were neutered; 24% had no special training, Data S1) were recruited from volunteers of the Family Dog Project database in Budapest, Hungary. Of these 43 were mixed-breeds, 31 border collies, 23 German shepherd dogs, 19 golden retrievers, 15 Labrador retrievers, 8 Malinois, 4 Hungarian vizslas, 3 Groenendaels, 3 standard poodles, 2 beagles, 2 tervuerens, 2 cocker spaniels, an Airedale terrier, bouvier des Flandres, bulldog, Cavalier King Charles spaniel, Doberman, German pointer, Irish setter, Perro de Presa Canario, kelpie, Maltese, mudi, rottweiler, Shetland sheepdog, Siberian husky, and Yorkshire terrier. Dogs categorized as trained (76% of the sample) participated in one or more of the following activities: obedience, agility, assistance, therapy, herding, guarding, detection, dog dancing, and search and rescue. Due to technical difficulties, the behavioral test was missing for four dogs, and, therefore, they were excluded from the analysis (Data S1).

### 2.2. Behavioral Test Protocol

**Figure 1:**
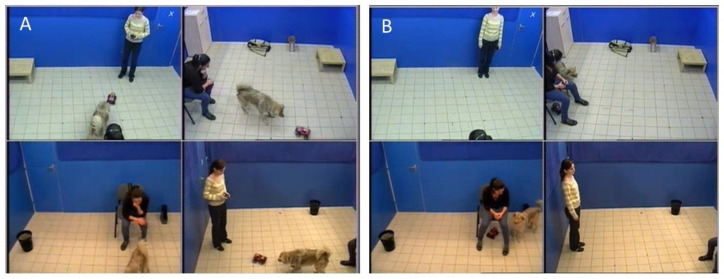
Scenes from the behavior test captured from four angles: (**A**) active phase (the toy car is moving) and (**B**) passive phase (the toy car is motionless under the chair). Written consent for publication was obtained from the individuals depicted.

All dogs were tested in an unfamiliar room (3 × 6 m^2^) at the Department of Ethology. The room contained various objects, including a chair for the owner, a large bag filled with books, an empty bin, a small table, a file folder, a paper box, another bag, and a chest of drawers where relevant experimental items were stored. Four cameras, one in each corner of the room, recorded all testing sessions. During testing, dogs were off-leash and free to move around the room (Figure 1).

Before the experiment began, dogs were familiarized with the experimenter for at least ten minutes to ensure that the experimenter was not perceived as a completely unfamiliar individual during the testing.

To assess dogs’ behavior in response to an ambiguous stimulus, the experimenter entered the room, retrieved a remote-controlled car from the drawer, placed it on the floor, and navigated it toward the dog for 20 s (active phase). Afterward, the experimenter stopped the car under the owner’s chair, and the dog’s behavior was observed and recorded for an additional 40 s (passive phase). A sample video is available here: https://youtu.be/2ZWGiLcWmVw, and as Video S1.

From the video recordings, we analyzed the frequency of gazes directed at the owner and the experimenter during both phases. As gazing events were typically brief, their occurrence provided more precise information than their duration. Additionally, we scored dogs’ approach behavior toward the car, assigning scores as follows: (2): the dog approached the car during the active phase; (1): the dog approached the car during the passive phase; (0): the dog did not approach the car during either phase.

### 2.3. Questionnaire

Before testing, owners filled in a survey and provided data about their dogs (breed, age, sex, neutered status, and training experience) and answered three additional questions to rate the emotional comfort provided by their companions on a Likert scale 1–5, expressing how much the respondent agrees with the statement (1): disagree, (5): agree strongly). The emotional comfort scale included three questions: (1) my dog enables me to love somebody; (2) my dog makes me feel loved; (3) my dog provides me with more companionship than anyone else. We also applied the Budapest Canine Personality Survey, where owners had to rate their dogs on a five-point scale on 17 items [36]. The ratings provide scores for four main traits: liveliness, confidence, aggressiveness (both toward dogs and humans), and attachment to their owners (Table S1).

### 2.4. Statistical Analysis

We used SPSS v26 for the statistical analysis. To assess the inter-observer reliability of the scoring, ten videos were coded by two observers. The inter-rater reliability of the variables was analyzed using a two-way random intraclass correlation, looking for absolute agreement between average measures. The reliabilities were satisfactory (both ICCs > 0.7, *N* = 10). The internal consistency of the three emotional comfort questions was tested with Cronbach’s alpha, and it was found to be good (0.746); therefore, we calculated a mean from the three scores and labeled the sum score as “emotional comfort”.

Two-step cluster analysis with Akaike’s Information Criterion was used for grouping the dogs based on the gazing at the owner and experimenter variables with automatic determination of the number of clusters. Within each group, we compared the frequency of gazing at the owner vs. gazing at the experimenter with a Wilcoxon signed-rank test and investigated the correlation between the two variables with a Spearman’s rho test. Between groups, we compared the variables with Kruskal–Wallis tests with pairwise comparisons (as included in the SPSSv29 software, which corrects for multiple comparisons with Bonferroni correction).

Only the liveliness score was normally distributed; therefore, we investigated how the clusters differ as a function of age, personality, and emotional comfort, using Kruskal–Wallis tests. Finally, we tested whether the clusters differed in sex, neutered status, purebred status, and breed category. The breed categories included five groups with a sample size of at least 15: mixed-breed, border collie, German shepherd dog, golden retriever, labrador retriever, training status, and approaching the car variables with Chi-square tests.

## 3. Results

### 3.1. Difference Between Dogs in How They Look at Human Partners (Hypothesis 1)

**Figure 2:**
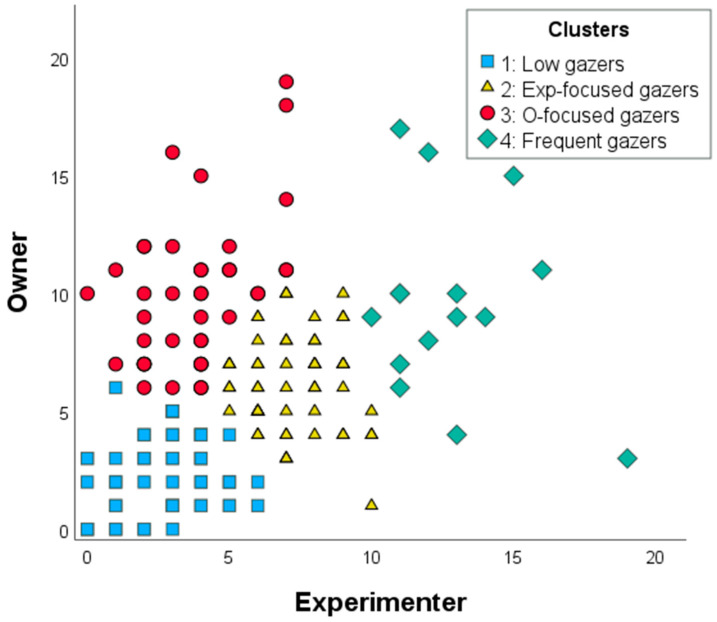
Scatter plot of the number of gazes at the owner and the experimenter variables, colored by clusters.

Based on the frequency of gazing toward the owner and the experimenter, cluster analysis identified four distinct clusters (Table 1). Approximately one-third of the dogs were categorized as “low gazers”, exhibiting minimal gazing behavior, typically only 2–3 gazes during the 1 min experiment, with no clear preference for either the owner or the experimenter. Another third of the dogs were classified as “experimenter-focused gazers”, directing significantly more gazes toward the unfamiliar experimenter (6–7 gazes) than toward their owner. In contrast, the “owner-focused gazers”, comprising about one-quarter of the sample, looked more frequently at their owner than the experimenter, with gaze frequencies ranging from four to ten. A smaller subset of dogs (9%) fell into the “frequent gazers” category. These dogs gazed intensively at both the owner and the experimenter, accumulating 10–12 gazes/person in total during the experiment, with no clear preference for one partner over the other. For more details about the clusters, see Supplementary Materials (Tables S2 and S3, Figure 2).

### 3.2. Differences Between the Clusters as a Function of Training Status, Age, Sex, Neutered Status, Purebred Status, Breed Categories, Four Personality Traits, Emotional Comfort, and Approaching the Car (Hypotheses 2–8)

Age, confidence, attachment, and the distribution of sex, neutered status, purebred status, training status, and approaching were not related to the cluster membership (all *p* > 0.05; see Supplementary Materials Tables S4 and S5).

Regarding breed categories, we found that 56.5% of German shepherd dogs were in cluster 1 (low gazers), in contrast to golden retrievers, where this number was only 5.3% (χ^2^ = 21.062, df = 12, *p* = 0.049).

**Figure 3:**
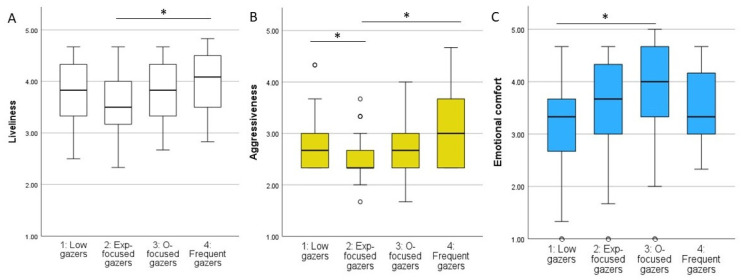
(**A**) Liveliness, (**B**) aggressiveness, and (**C**) emotional comfort across clusters (1 to 4). * indicates significant differences (*p* < 0.05). In the boxplots, the rectangular box represents the middle 50% of the data (interquartile range, IQR). The horizontal line represents the median. Whiskers indicate the spread of the non-outlier data. Outliers are shown as small circles.

Liveliness and aggressiveness personality traits, as well as emotional comfort, differed between the four clusters (KW = 10.633, *p* = 0.014; KW = 13.325, *p* = 0.004; KW = 9.918, *p* = 0.019; respectively; Figure 2). Post hoc pairwise comparisons between the clusters indicated that dogs in cluster 2 (experimenter-focused gazers) were reported to be less lively than those in cluster 4 (Figure 3A, Table S4). These dogs were also reported to be less aggressive than those in clusters 1 (low gazers) and 4 (frequent gazers) (Figure 3B, Table S7). Dogs in cluster 3 (owner-focused gazers) provided more emotional comfort than those in cluster 1 (low gazers) (Figure 3C, Table S8).

## 4. Discussion

Our observations revealed that in the ambiguous situation, dogs could be grouped into four distinct clusters based on their gazing frequency and preferences. These clusters differed not only in the overall frequency of gazing but also in whether dogs showed a preference for gazing at their owner, the unfamiliar person (experimenter), or both. This clustering highlights the variability in dogs’ social engagement strategies and their differential use of gazing as a form of communication. We assumed that these differences may reflect individual variation in age, sex, prior experiences such as training status, approach behavior, personality, and the emotional comfort the dog provides for the owner. The results only partially supported our expectations. We detected differences in gazing behavior between two breeds (more than half of the German shepherd dogs were low gazers in contrast to golden retrievers), and found that two personality traits (liveliness and aggressiveness) and the emotional comfort the dog offers the owner differed between the clusters.

The finding that when an owner and another person are present in an experimental setup, some dogs tend to gaze more toward one individual than the other is not entirely new. For instance, Marshall-Pescini et al. [12] observed that agility dogs focused more specifically on their owner and not the experimenter in an unsolvable task. Similarly, D’Aniello et al. [37] reported that water rescue dogs gazed predominantly at their owner. In line with these findings, we found that approximately one-third of the dogs displayed a clear preference for gazing at the owner. However, in contrast to earlier studies, this behavior was not associated with training status in our experiment, which can be explained by the fact that training in this specific group was highly diverse; therefore, with fewer training variables, a different result might have emerged.

The lack of a relationship between training and gazing frequency in this study might be explained by several factors. First, as Carballo et al. [26] suggested, “training experience” is often too broadly defined, making it an imprecise variable. Moreover, there can be substantial overlap in the training experiences of working dogs (e.g., assistance or therapy dogs) and companion dogs. Additionally, some studies [22,23,38] have reported that training type does not necessarily affect gazing frequency, suggesting that other factors may play a more prominent role in driving this behavior.

Regarding age effects, in contrast to some studies [19,20,24] but consistent with others [25,28], we did not observe an effect of age in our study. This discrepancy could be attributed to the different contexts used: in our test, dogs encountered an ambiguous stimulus, whereas previous studies focused on unsolvable tasks or eye-contact establishment. Another possible explanation is the relatively small proportion of old dogs in our sample (22.2%), which may have limited the statistical power to detect age-related differences.

Similarly, while previous studies have shown that females tend to exhibit more social behaviors, such as gazing, in various tasks [20,39,40], we found no effect of sex on gazing frequency. This lack of a sex effect may reflect the ambiguous nature of the situation we created in the experiment, which may not have elicited the same behavioral tendencies observed in other contexts.

Genetic factors have also been implicated in gazing behavior, with studies showing that basal breeds or independent breeds tend to gaze less than breeds selected for cooperative work with humans [24,41,42] and that gazing is linked to D4-type dopamine receptor (*DRD4*) [40] and oxytocin receptor (*OXTR*) gene polymorphisms [43]. In this study, due to sample size limitations, we could compare only five breed groups, with only mixed-breed dogs categorized as independent [44]; the other four are all cooperative (border collie, German shepherd dog, golden retriever, Labrador retriever). We found that contrary to what their name suggests, German shepherd dogs did not behave as typical shepherd dogs selected for visual cooperation, such as border collies, because they were more frequently categorized as low gazers than golden retrievers.

Interestingly, our analysis revealed that personality traits—specifically liveliness and aggression—and the level of emotional comfort dogs offer their owners significantly influenced gazing behavior. Low gazers scored higher for aggression than experimenter-focused gazers. This aligns with the suggestion in [45] that aggression is negatively related to social interest, potentially explaining the reduced gazing behavior in this group. However, frequent gazers were also reported to be more aggressive than experimenter-focused gazers, suggesting that for these dogs (only nine percent of the sample) high gazing frequency was related to high anxiety in the ambiguous situation [31]. Another characteristic of frequent gazers is that they gazed more at the experimenter than at the owner. These dogs were also characterized by the highest aggression scores. While aggression is often associated with reduced social interest, as discussed earlier, it is also linked to fear of strangers [46]. The increased gazing at the experimenter in this cluster may, therefore, reflect heightened fear or vigilance in response to the unfamiliar person.

Owners of owner-focused gazers reported that their dogs provided higher levels of emotional comfort. This pattern is consistent with previous findings that dogs encountering a potentially threatening or ambiguous stimulus tend to gaze more at their owner, seeking comfort and reassurance [32], indicating that dogs in this cluster likely perceived their owners as a source of emotional support, a “safe haven” [47], or they have learned that this is an owner expectation.

## 5. Conclusions

Our findings demonstrate that dogs can be categorized based on their gazing patterns linked to specific personality traits and their relationship with their owners. To deepen our understanding of how individual personality variables influence gazing behavior in family dogs, future studies could benefit from incorporating broader personality assessment methods, such as questionnaires measuring traits like independence, playfulness, and impulsivity (e.g., [48,49]). Additionally, more specific training categories could be explored; for instance, the training of hunting dogs often includes exercises focused on eye contact, which presumably affects gazing behavior in certain contexts.

Further research should also include physiological measures and detailed behavioral analyses to investigate how curiosity, fear, or neutrality affect gazing behavior. These analyses may help uncover the factors driving differences in dogs’ social engagement and communication strategies, such as seeking comfort, permission, approval, or guidance in challenging situations. Extending the research to more varied scenarios—such as social play, exploration, or neutral settings—could provide new insights into the flexibility of gazing behavior and how variables like relationship quality and the dog’s emotional state influence it.

## Figures and Tables

**Table 1:** The mean frequency of gazes at the owner (O) and the experimenter (E) ± SD for each cluster and the statistical values.

Cluster	N	% of Total	Looking at the O ± SD	Looking at the E ± SD	Comparing Looking at the Partners
1: Low gazers	55	32.5%	2.4 ± 1.6	2.8 ± 1.6	O = E, W = 1.484, *p* = 0.138
2: E-focused gazers	56	32.7%	6.2 ± 2.1	7.3 ± 1.5	O < E, W = 2.606, *p* = 0.009
3: O-focused gazers	41	24.0%	10.0 ± 3.1	3.8 ± 1.8	O > E, W = 5.590, *p* < 0.001
4: Frequent gazers	15	8.8%	9.6 ± 4.0	12.8 ± 2.4	O < E, W = 2.144, *p* = 0.032

## Data Availability

Data are available as Supplementary Materials.

## Supplementary Materials

The following supporting information can be downloaded at https://www.mdpi.com/article/10.3390/ani15040483/s1, Table S1. Items of the Budapest Canine Personality Survey (BCPS); Table S2. Pairwise comparisons of the Looking at the owner variable across clusters; Table S3. Pairwise comparisons of the Looking at the experimenter variable across clusters; Table S4. Comparing the clusters as a function of age, Budapest Canine Personality Survey (BCPS) traits, and Emotional comfort scale; Table S5. Comparing the clusters with Chi square tests; Table S6. Pairwise comparisons of the Liveliness variable across clusters; Table S7. Pairwise comparisons of the Aggressiveness variable across clusters; Table S8. Pairwise comparisons of the Emotional comfort variable across clusters; Data S1, and Video S1. Dogs’ gazing behavior toward humans is related to their personality, and emotional comfort.
