# Supplementary material for: Dogs’ Gazing Behavior to Humans Is Related to Their Liveliness, Aggressiveness, and the Emotional Comfort They Provide
Source: Animals (Basel). 2025 Feb 8;15(4):483. doi: 10.3390/ani15040483 (PMC11851834; doi:10.3390/ani15040483)
Supplement: Supplementary file 1 [file animals-15-00483-s001.zip › SM_Kubinyi_Sommese_etal_2025_gaze.pdf]

Supplementary Materials

Dogs' gazing behavior to humans is related to their liveliness, aggressiveness, and the emotional comfort they provide

Eniko Kubinyi [1,2,#,*], Andrea Sommese [1,#], Márta Gácsi [1,3], Ádám Miklósi [1,3]

[1] Department of Ethology, Eötvös Loránd University, 1117. Budapest, Hungary
[2] MTA-ELTE "Momentum" Companion Animals Research Group, 1117, Budapest, Hungary
[3] HUN-REN-ELTE Comparative Ethology Research Group, Budapest, Hungary
# Equal contribution
* Correspondence: Eniko Kubinyi, eniko.kubinyi@ttk.elte.hu

Table S1. Items of the Budapest Canine Personality Survey (BCPS, [1])

		5	4	3	2	1	
1 [a]	Very playful	☐	☐	☐	☐	☐	Not at all playful
2 [b]	Confident	☐	☐	☐	☐	☐	Nervous
3 [b]	Relaxed in unfamiliar situations	☐	☐	☐	☐	☐	Timid in unfamiliar situations
4 [d]	Strongly attached	☐	☐	☐	☐	☐	Not at all attached
5 [a]	Highly excitable, impulsive	☐	☐	☐	☐	☐	Calm, placid
6 [d]	Obedient	☐	☐	☐	☐	☐	Disobedient
7 [a]	Active, energetic	☐	☐	☐	☐	☐	Inactive, lazy
8 [c]	Friendly	☐	☐	☐	☐	☐	Unfriendly
9 [d]	Intelligent	☐	☐	☐	☐	☐	Slow in thinking
10 [b]	Brave	☐	☐	☐	☐	☐	Timid
11 [a]	Curious	☐	☐	☐	☐	☐	Not curious
12 [b]	Can handle being alone	☐	☐	☐	☐	☐	Cannot handle being alone
13 [d]	Requires lots of care and attention	☐	☐	☐	☐	☐	Does not require lots of care and attention
14 [a]	Pushy	☐	☐	☐	☐	☐	Not pushy
15 [a]	Highly possessive	☐	☐	☐	☐	☐	Not at all possessive
16 [c]	Aggressive with unfamiliar people	☐	☐	☐	☐	☐	Not aggressive with unfamiliar people
17 [c]	Aggressive with unfamiliar dogs	☐	☐	☐	☐	☐	Not aggressive with unfamiliar dogs

[a] Liveliness scale
[b] Confidence scale
[c] Aggressiveness scale (Item 8 is reverse-scored.)
[d] Attachment scale

Comparing the Looking at the owner and Looking at the experimenter variables between the clusters
According to the between cluster comparisons, clusters differed in the Looking at the Owner variable (KW=112.903, p<0.001), except Cluster 2-4 and Cluster 3-4 (Table S2). Similarly, clusters differed in the Looking at the Experimenter variable (KW=118.647, p<0.001), except Cluster 1-3 (Table S3).

Table S2. Pairwise comparisons of the Looking at the owner variable across clusters. Significant differences are highlighted in bold.

Sample 1-Sample 2	Test Statistic	Std. Error	Std. Test Statistic	Sig.	Adj. Sig.[a]
1: Low gazers-2: Exp-focused gazers	-55.977	9.148	-6.119	<.001	**.000**
1: Low gazers-4: Frequent gazers	-90.258	14.036	-6.430	<.001	**.000**

1: Low gazers-3: O-focused gazers	-99.032	9.942	-9.961	<.001	**.000**
2: Exp-focused gazers-4: Frequent gazers	-34.281	14.009	-2.447	.014	.086
2: Exp-focused gazers-3: O-focused gazers	-43.056	9.904	-4.347	<.001	**.000**
4: Frequent gazers-3: O-focused gazers	8.775	14.540	.603	.546	1.000

Each row tests the null hypothesis that the Sample 1 and Sample 2 distributions are the same.
Asymptotic significances (2-sided tests) are displayed. The significance level is .050.
a. Significance values have been adjusted by the Bonferroni correction for multiple tests.

Table S3. Pairwise comparisons of the Looking at the experimenter variable across clusters. Significant differences are highlighted in bold.

Sample 1-Sample 2	Test Statistic	Std. Error	Std. Test Statistic	Sig.	Adj. Sig.[a]
1: Low gazers-3: O-focused gazers	-16.896	9.928	-1.702	.089	.533
1: Low gazers-2: Exp-focused gazers	-77.187	9.135	-8.450	<.001	**.000**
1: Low gazers-4: Frequent gazers	-116.348	14.017	-8.301	<.001	**.000**
3: O-focused gazers-2: Exp-focused gazers	60.291	9.891	6.096	<.001	**.000**
3: O-focused gazers-4: Frequent gazers	-99.452	14.520	-6.849	<.001	**.000**
2: Exp-focused gazers-4: Frequent gazers	-39.161	13.990	-2.799	.005	.031

Each row tests the null hypothesis that the Sample 1 and Sample 2 distributions are the same.
Asymptotic significances (2-sided tests) are displayed. The significance level is .050.
a. Significance values have been adjusted by the Bonferroni correction for multiple tests.

Correlations between Looking at the owner and Looking at the experimenter variables within the clusters

Gazing at the owner or at the experimenter did not correlate in Cluster 1 (rho=0.167, p=0.224), Cluster 2 (rho=-0.033, p=0.810) and Cluster 4 (rho=-0.061, p=0.830) but did so in Cluster 3 (rho=0.358, p=0.022).

Table S4. Comparing the clusters as a function of age, Budapest Canine Personality Survey (BCPS) traits, and Emotional comfort scale (Kruskal Wallis tests, df=3). Significant differences are highlighted in bold.

Variable	KW	Sig.
Age	2.005	0.571
BCPS Liveliness	10.633	**0.014**
BCPS Confidence	1.552	0.670
BCPS Aggressiveness	13.325	**0.004**
BCPS Attachment	3.018	0.389
Emotional comfort	9.918	**0.019**

Table S5. Comparing the clusters with Chi-square tests. The significant difference is highlighted in bold.

Variable	Chi-square	df	Sig.
Purebred status	0.417	3	0.937
Breed category	21.062	12	**0.049**
Sex	4.750	3	0.191
Neutered status	1.079	3	0.782
Training status	3.685	3	0.298
Approaching the car	1.501	6	0.959

Table S6. Pairwise comparisons of the Liveliness variable across clusters. Significant difference is highlighted in bold.

Sample 1-Sample 2	Test Statistic	Std. Error	Std. Test Statistic	Sig.	Adj. Sig.[a]
2: Exp-focused gazers-1: Low gazers	17.141	8.704	1.969	.049	.293
2: Exp-focused gazers-3: O-focused gazers	-22.651	9.390	-2.412	.016	.095
2: Exp-focused gazers-4: Frequent gazers	-36.249	13.092	-2.769	.006	**.034**
1: Low gazers-3: O-focused gazers	-5.510	9.430	-.584	.559	1.000
1: Low gazers-4: Frequent gazers	-19.107	13.121	-1.456	.145	.872
3: O-focused gazers-4: Frequent gazers	-13.597	13.586	-1.001	.317	1.000

Each row tests the null hypothesis that the Sample 1 and Sample 2 distributions are the same.
Asymptotic significances (2-sided tests) are displayed. The significance level is .050.
a. Significance values have been adjusted by the Bonferroni correction for multiple tests.

Table S7. Pairwise comparisons of the Aggressiveness variable across clusters. Significant differences are highlighted in bold.

Sample 1-Sample 2	Test Statistic	Std. Error	Std. Test Statistic	Sig.	Adj. Sig.[a]
2: Exp-focused gazers-3: O-focused gazers	-21.305	9.013	-2.364	.018	.109
2: Exp-focused gazers-1: Low gazers	22.364	8.354	2.677	.007	**.045**
2: Exp-focused gazers-4: Frequent gazers	-38.670	12.567	-3.077	.002	**.013**
3: O-focused gazers-1: Low gazers	1.059	9.052	.117	.907	1.000
3: O-focused gazers-4: Frequent gazers	-17.365	13.041	-1.332	.183	1.000
1: Low gazers-4: Frequent gazers	-16.306	12.595	-1.295	.195	1.000

Each row tests the null hypothesis that the Sample 1 and Sample 2 distributions are the same.
Asymptotic significances (2-sided tests) are displayed. The significance level is .050.
a. Significance values have been adjusted by the Bonferroni correction for multiple tests.

Table S8. Pairwise comparisons of the Emotional comfort variable across clusters. Significant difference is highlighted in bold.

Sample 1-Sample 2	Test Statistic	Std. Error	Std. Test Statistic	Sig.	Adj. Sig.[a]
1: Low gazers-4: Frequent gazers	-8.093	12.798	-.632	.527	1.000
1: Low gazers-2: Exp-focused gazers	-12.397	8.015	-1.547	.122	.732
1: Low gazers-3: O-focused gazers	-28.076	8.971	-3.130	.002	**.010**
4: Frequent gazers-2: Exp-focused gazers	4.304	12.599	.342	.733	1.000
4: Frequent gazers-3: O-focused gazers	19.982	13.227	1.511	.131	.785
2: Exp-focused gazers-3: O-focused gazers	-15.678	8.684	-1.805	.071	.426

Each row tests the null hypothesis that the Sample 1 and Sample 2 distributions are the same.
Asymptotic significances (2-sided tests) are displayed. The significance level is .050.
a. Significance values have been adjusted by the Bonferroni correction for multiple tests.

References

1. Wan, M.; Kubinyi, E.; Miklósi, Á.; Champagne, F. A Cross-Cultural Comparison of Reports by German Shepherd Owners in Hungary and the United States of America. *Appl. Anim. Behav. Sci.* **2009**, *121*, 206–213, doi:10.1016/j.applanim.2009.09.015.
